# The impact of physician associates in primary care in the UK: a systematic review

**DOI:** 10.3399/BJGPO.2025.0026

**Published:** 2025-12-19

**Authors:** Susan Willacy, Umaiyal Ravindran, Oyinlola Oyebode

**Affiliations:** 1 Queen Mary University of London, London, UK; 2 Community Dental Service Community Interest Company, Essex, UK; 3 Wolfson Institute of Population Health, Queen Mary University of London, London, UK

**Keywords:** primary health care, systematic review, workforce

## Abstract

**Background:**

The UK healthcare system has a growing workforce crisis, which is felt especially acutely in primary care. A prospective solution is the use of physician associates (PAs). In recent times, this has generated some controversy. There is a sparsity of synthesised evidence around the use of PAs in the UK, particularly their implementation in primary care.

**Aim:**

To look at the impact PAs have on workload, safety, efficacy, and cost-effectiveness in delivering UK primary care.

**Design & setting:**

Systematic review of peer-reviewed literature, including qualitative and quantitative studies of PAs, in UK primary care.

**Method:**

Cochrane and Preferred Reporting Items for Systematic reviews and Meta-Analyses (PRISMA) guidelines were followed. PubMed, Embase, the Cochrane Library, Web of Science, and CINAHL were searched from 2011–2024. Covidence was used for data management. Narrative synthesis was performed based on the four primary aims.

**Results:**

Sixteen studies were deemed to meet the inclusion criteria for data extraction and synthesis. Thirteen commented on workload, eight commented on safety, 13 on efficacy, and eight studies discussed cost-effectiveness. Results showed that PAs were considered clinically safe but impacts on workload and efficacy were less clear. Cost-effectiveness assessment was limited by inability to calculate full costs or benefits.

**Conclusion:**

This review found that there is limited evidence available in the published literature to demonstrate the impact of PAs in primary care. While there were some positive studies, a clear need for further research was demonstrated. An additional pathway to explore, comparing PAs with the non-GP primary care workforce, was also noted.

## How this fits in

There is a sparsity of published evidence around the efficacy of physician associates (PAs) and particularly the economic argument for their use. With the growing consideration of use of PAs within UK primary care, the increased media interest in PAs and the associated controversy, more current, geographically specific evidence will be essential for future workforce planning and defining the role of PAs within primary care.

## Introduction

There has been a rising workforce crisis across the UK healthcare system for many years now, and this has been felt especially acutely in primary care.^1,2^

Between September 2015 and July 2022 there was a decrease of 6.3% of the GP workforce.^3^ Modelling suggests that with current trends there could be a shortfall of 8800 GPs by 2030–2031.^3^

A potential solution is the expansion of other professions to undertake some of the work that has traditionally been part of a core function of GPs. A newer example of these professional roles are the physician associates (PAs). PAs are healthcare professionals who have completed a generalist medical education via a 2-year postgraduate degree, and work under the supervision of doctors but with a degree of autonomy. While well established in the US (where they are known as physician assistants), they have only been working in the UK since 2003.^4^ There are approximately 3250 PAs working within the NHS, with over half working within primary care, and in an effort to manage workforce pressures, the NHS plans to increase this total number to 10 000 by 2036–2037.^5^

The use of PAs in both primary and secondary care has attracted controversy in recent times.^6–9^ During the writing of this review, in September 2024, the Royal College of General Practitioners (RCGP) voted against the inclusion of PAs working in general practice, a change on their previous standpoint.^10^ Similar guidance has been produced by the British Medical Association (BMA),^11^ and is being considered by both the royal surgical and medical colleges.^12,13^

While there are some systematic reviews on PAs,^14–17^ which suggest that there was potential for PAs to add value to primary care teams, regulations (and lack of prescribing and other responsibilities) may limit the scope of their role. Just one review was available to address the specific issue of PAs impact on UK primary care,^15^ which included studies published up until 2020 and was completed by a single study author, which suggests the possibility that there is additional and potentially more recent research that may not have been identified and synthesised in that review.

### Objectives

The primary objective of this study is to investigate from the available recent research and evidence both measures of, and the perspectives of stakeholders, on four key outcomes:

- Safety, which was chosen owing to both the importance to clinical practice and also because of recent concerns raised within the media. We defined this using NHS England’s definition *'of the avoidance of unintended or unexpected harm to people during the provision of health care*'.^18^
- Workload, which was chosen owing to being noted as a primary driver of the GP workforce crisis.^3^ We defined this by considering the impact PAs had on the overall workload of the practice.
- Efficacy, this was considered to be important based on the initial literature review, indicating there may be some misalignment with expected roles and how this worked practically. We looked for assessment of this in our data extraction.
- Cost-effectiveness, this was deemed important based on the consistent financial constraints within health systems. We looked for direct and indirect assessments of this in the literature.

## Method

The review was registered with PROSPERO on 24 June 2024 (reference: CRD42024568061) and is reported following Cochrane and Preferred Reporting Items for Systematic reviews and Meta-Analyses (PRISMA) guidelines.^19^ A PICOS framework was used to guide our search strategy as per Table 1.

### Information sources

We searched the following electronic bibliographic databases: PubMed; Embase; the Cochrane Library; Web of Science (Science and Social Science Citation Index); and CINAHL.

Searches were developed over several iterations and the final search strategy was confirmed after input from an academic librarian. Additional eligible studies were sought via hand screening of reference lists, professional publications, and via citation alerts after the searches were complete. During the development of the search strategy, it became clear that the number of studies would likely be small. Consequently, search terms were developed to be as sensitive as possible to capture all UK primary care studies on PAs. The researchers then assessed for safety, workload, efficacy, and cost-effectiveness outcomes within the wider pool of identified studies.

The searches were re-run (1 November 2024) just before the final analyses but no further studies were retrieved for inclusion (Table 1).

**Table 1.** PICOS framework

**Participants:** studies of UK primary care or general practice
**Intervention:** placement of physician associates in the UK
**Comparators:** practices without physician associates or before their placement
**Outcomes:** measures of safety, workload, efficacy, and cost-effectiveness as well as the perspectives of stakeholders on safety, workload, efficacy, and cost-effectiveness
**Study design:** RCTs and controlled trials were included. However, it was not anticipated many RCT or controlled trials would have been completed and therefore observational and qualitative studies were also eligible. Economic analyses were eligible.
Excluded: non-primary care studies, non-UK-based studies, studies before 2011 (owing to limited numbers of physician associates working in UK primary care until this time). Non-English language literature. Editorials and letters, narrative or systematic reviews.

RCTs = randomised controlled trials

### Eligibility criteria

#### Study selection

All identified studies underwent two stages of screening with two independent researchers: title and abstract screening where all studies deemed eligible by one researcher were passed to full-text screening, and full-text screening as per the eligibility criteria. Any disagreements at full-text screening were discussed and a consensus agreed on. Covidence was used for study screening and record management

#### Data extraction process

Two researchers independently extracted the data.

A Covidence database entry form was used with specific extraction fields based on a preliminary synthesis detailed below.

This included the research question or purpose, methods, date of data collection, possible conflicts of interests, study design, outcomes discussed, participants characteristics and sample size, inclusion and exclusion criteria, analysis methods, findings of interest, and authors' conclusions.

This was an iterative process as publications were reviewed, with entry form trialled on two studies before agreeing on the extraction database. Once complete, data were collated and summarised based on the research questions, influenced by the literature identified.

#### Data items

In many papers the outcomes we assessed for were the main outcomes sought by the studies and were clearly defined; however, in those that were more ambiguous researchers agreed in which outcome grouping the results would sit between them.

All relevant results were included in the outcome reporting from each study. Owing to the primarily qualitative nature of the studies, no summary effect measures could be synthesised for this review and, instead, a narrative synthesis was performed.

#### Quality assessment

Quality assessment was undertaken by two researchers independently at time of data extraction. The Mixed Methods Appraisal Tool (MMAT), adapted into the Covidence quality assessment template, was used to critically appraise the quality of the studies, which is a validated tool for most common types of study.^20^

## Results

### Description of included papers

**Figure 1.**
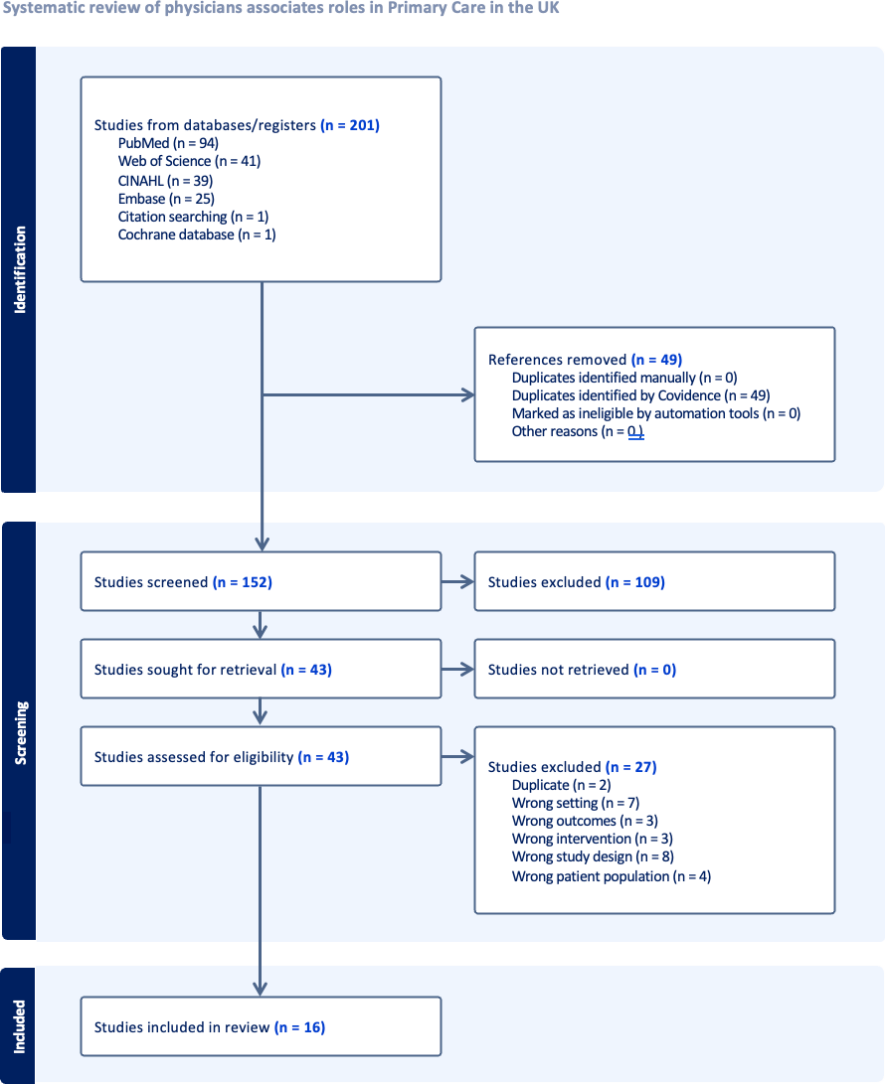
Preferred Reporting Items for Systematic reviews and Meta-Analyses (PRISMA) flowchart from search to included studies

The initial search identified 152 unique papers of which 43 were put forward to full-text review. Following this, 16 were deemed to meet the inclusion criteria for data extraction and inclusion in the review (Figure 1).

Table 2 shows an examples search strategy. Table 3 demonstrates that of the studies that were eligible for data extraction, the majority (9/16) were qualitative in nature, with other mixed method (2/16), and observational studies (5/16) making up the rest.

**Table 2.** Example search strategy

Example PubMed strategy
((("physician assistants") OR (((("Physicians assistant") OR ("physician associate")) OR ("medical associate"profession*)) OR ("Physician Assistants"[Mesh]))) AND (((("England") OR (("united Kingdom"))) OR ("United Kingdom"[Mesh])) OR ("England"[Mesh]))) AND ((((((("general practi*") OR ("delivery of healthcare")) OR ("primary care")) OR ("Delivery of Health Care"[Mesh])) OR ("General Practitioners"[Mesh])) OR ("General Practice"[Mesh:NoExp])) OR ("Access to Primary Care"[Mesh] AND "Primary Health Care"[Mesh]))

**Table 3.** Study characteristics

Study	Study aim	Methods	Dates of data collection	Participants
Agarwal *et al*, 2021^34^	How can clinical supervisors support and supervise PAs in primary care? What factors should be considered? What are the main components of a PA clinical supervisor role?	Qualitative research	Not clear	20 primary care PAs within 16 practices. 14/20 responded
Krachler *et al*, 2023^21^	To explore the use of different forms of power by GPs in engaging with PAs	Qualitative research	2019–2021	20 GP partners, PAs, and stakeholders
Nelson *et al*, 2019^22^	To compare how three non-medical roles were established in general practice, identify barriers, and explore impacts or unintended consequences	Qualitative research	2017–2018	9 service or training leads, 18 trainees or practitioners, 11 GP practice staff
Drennan *et al*, 2014^23^	How are PAs deployed, and what is their impact on patients' experience, practice organisation, and costs? Factors supporting or inhibiting inclusion of PAs	Mixed-methods research	2011–2012	12 practices, patient records (2086) interviews and patient surveys (539), video consultation
Drennan *et al*, 2011^36^	To study the motivation of GPs and practice managers employing PAs and factors sustaining their employment	Observational study	2009	13 GPs, 3 practice managers from PA-employing practices
Drennan *et al*, 2012^31^	What is the extent of PA employment in primary care in England and their contribution to patient care?	Qualitative research	2010	16 PAs
Drennan *et al*, 2015^24^	To compare outcomes and costs of same-day consultations by PAs and GPs	Observational study	2011–2012	2086 consultation records
de Lusignan *et al*, 2016^25^	Investigating consultation quality of PAs versus GPs, focusing on safety and practitioner identification	Observational study	2012	62 videos of consultations from 12 practices
Drennan *et al*, 2017^32^	How does a new professional group fit with established professions in primary care?	Mixed-methods research	2011–2012	25 senior individuals in national and regional groups, 39 staff in primary care centres
Halter *et al*, 2017^26^	To investigate patients’ perspectives on consulting with PAs in general practice	Qualitative research	2011–2012	30 patients
Jackson *et al*, 2017^27^	To investigate barriers and facilitators to integrating PAs into the general practice workforce	Qualitative research	2015	51 GPs, ANPs, and patients
Hoggins *et al*, 2018^29^	To provide insights into the experiences of PA students and primary care staff during educational placements in the UK	Qualitative research	2016	8 PAs and 6 primary care staff
Halter *et al*, 2018^33^	To develop a case-mix classification system and test its impact on patient outcomes analysis for PAs versus GPs consultations	Observational study	2011–2012	2086 consultations, 12 volunteer practices
Hoskin *et a*l 2020^30^	How can GPs be encouraged to use PAs effectively and integrate them into the primary care team?	Qualitative research	2020	8 PAs and their supervising GPs
Cottrell *et al*, 2021^28^	How could an internship support PA integration into primary care teams? What is the acceptability of PA interns in primary care?	Qualitative research	2017–2018	10 PAs, 8 practices, 165 patients
Gibson *et al*, 2023^48^	To investigate factors motivating GP practices to employ new roles and the role of financial incentives	Observational study	2019	1205 practice managers

ANPs = advanced nurse practitioners. PAs = physician associates

Of the studies in this paper, 10 out of 16 were judged either low or low to medium risk of bias as noted in Table 4, with only one ranking medium to high. This was largely owing to small sample sizes (which is difficult to avoid given the small pool of PAs available during the studies), poor response rates, or unclear exclusion criteria.

**Table 4.** Quality and bias assessment

Study ID (first author)	Risk of bias High, medium, or low
**Agarwal 2021**	**Low:** response rate 70% (although small sample)
**Krachler 2023**	**Medium:** range of sources gathered data although raw data not shared
**Nelson 2019**	**Low:** appropriate sampling methods for study purpose
**Drennan 2014**	**Low:** comprehensive mixed-methods study, large overall sample size with clear inclusion criteria
**Drennan 2011**	**Medium:** response rate 15/22 practices, cannot tell if those responding differed from those not responding so may affect how representative they are
**Drennan 2012**	**Low:** response rate reported at 64% and while not clear whether there was difference in those responding to the survey or not, the aims of the study would be unlikely to be influenced by this
**Drennan 2015**	**Low:** large sample size with clear inclusion criteria
**de Lusignan 2016**	**Low:** clear inclusion criteria and blinded assessment tools
**Drennan 2017**	**Low to medium:** reasonable response rate to interviews (25/50 at macro level 39/48 at micro) but unclear if differences between those who responded and who did not
**Halter 2017**	**Low to medium:** clear inclusion criteria noted but all those who met the criteria were not accounted for in the methods section
**Jackson 2017**	**Low:** range of participants sampled and justified
**Hoggins 2018**	**Medium:** small sample and potential for sampling bias
**Halter 2018**	**Low:** a secondary analysis of pre-collected patient records
**Hoskin 2020**	**Medium:** unclear what response rate or composition of the survey was
**Cottrell 2021**	**Medium:** variable response rates over time and by component means sampling bias may be a possibility
**Gibson 2023**	**Medium to high:** 17% response rate, and authors noted practices included were larger and less deprived than the non-sample practices

### Study design

Of the 16 studies identified by the review, eight commented on safety, 13 on workload, 13 on efficacy, and eight looked at cost-effectiveness. The below synthesis is derived primarily from the authors results and conclusions. Details of data extracted from each study is available in Supplementary Table 1.

#### Safety

Eight of the studies reported on elements of safety whether directly or indirectly as part of their analysis.^21–28^ Studies with stakeholder interviews (*n* = 3), primarily with GPs, expressed some theoretical concerns around safety often centred around the prescribing limitations and the logistical patient flow problems that this causes:^21,22,27^

*'*[PAs] *are clinically very well-trained individuals … but they can’t prescribe. So you can’t let them do clinics independently.'* (GP)*^21^*

Other points of contention included the relative lack of experience of the PA in a primary care setting and the ability to meet the needs of the more complex patients:^27,28^

*'I don’t know how the physician associates have the experience to know what they don’t know.'* (GP)*^27^*

In contrast the studies with data on patient perspectives on PAs and safety (*n* = 3) found that patients' experiences were largely positive.^24,26,28^ Responders in Drennan *et al*'s 2014 study described trust in the PAs owing to demonstrated clinical competence, with participants feeling the consultations were very similar to that of one with a GP.^23^ In Halter *et al*’s 2017 study, patients described PAs as inspiring high trust and confidence fulfilling a role similar to that of a GP:^26^

*'Well they've* [the PAs] *never given a diagnosis that I didn't think was a good diagnosis, they've always given the right medicine in my opinion, it’s always worked. So I've never, ever had a problem, that’s why I feel confident with them. It’s as if you're seeing a doctor.'* (Patient)^26^

Of the papers that assessed PA consultations directly^23–25^ (*n* = 3; although two of these papers report data from the same study)^23,25^ all were deemed clinically safe. De Lusignan *et al* 2016 found when comparing GP and PA consultations using blinded raters that all consultations of both professions were considered safe. However, in overall consultation scores GPs rated higher across all domains, deemed significant in patient management and problem-solving.^25^

In Drennan *et al*’s 2015 analysis of patient consultations from both GP and PA records, the written records of the PA consultations were considered more systematic than the GP comparators.^24^

It was noted that there were patients who were unclear which professional they were consulting with even following the consultation.^23^ This confusion led to discontentment when realising that they were not being assessed by a GP. This has also been echoed in Cottrells *et al*’s 2021 study with 29% of the patients in their survey not realising they had seen a PA.^28^ This could cause safety issues in how patients may follow-up on health issues and their understanding of their own consultations.

#### Workload

Thirteen of the 16 studies commented on aspects that related to workload.

Positive views of workload were found in a number of studies (*n* = 5) with Drennan *et al*’s paper in 2014^23^ describing PAs as acceptable, effective, and efficient in complementing the work of GPs. Other studies^25,28–30^ considered there was a place for them within the primary care workforce, with many who have employed PAs being positive about the experience.

Drennan *et al* (2012)^31^ found in a survey of PAs that their primary workload was predominately same-day appointments and urgent consultations. In Drennan *et al* (2017)^32^ they also described PAs seeing entirely unselected and unfiltered patients:

*'He* [the PA] *sees a surgery of patients morning and afternoon every day, which are almost entirely unselected. We have selected out under ones because he is not trained for those, but other than that he sees the full range of problems that present.'* (GP*)* ^32^

Drennan *et al* further described aspects of the PA’s workload in another study in the same series,^24^ calculating that the PAs saw two patients for every three seen by the GPs, spending an average of 5.8 minutes longer per consultation.

A number of studies (*n* = 4) reported on the complexity of cases seen by PAs relative to GPs^21,22,24,33^, finding that PAs tended towards seeing the less complex patients. As noted by Drennan *et al* (2015)^24^ and Kracher *et al* (2023)^21^ receptionists or GPs may triage patients for PA consultations, which may explain how this occurs. De Lusignan *et al* (2016) described GPs as being more likely to see patients with multiple problems, with 44% of GP consultations having at least two problems compared with only 5% of PA consultations. Similarly, patients with chronic problems were found more likely to consult with a GP.^25^

Induction and supervision were looked at in three studies^28,30,34^ and seemed to be strongly linked to how experienced the PA was. In Hoskin *et al*’s 2020 paper on a preceptorship scheme for newly qualified PAs, results indicated that the initial outlay in supervision time was relatively high but this reduced over the first year.^30^ Direct supervision was described as including regular debriefs^30,34^ (ranging from once to twice daily to after every patient) reducing down over time to as required. Appointment duration also ranged down from 30-minute appointments to 15 minutes over time demonstrating increasing productivity of the PAs. In Krachler et al’s 2023 work^21^ concerns were raised about the amount of supervision and the impact this may have on the practice:

*'Our concern would be the balance of supervision time from our GPs at the moment and blocking people out to supervise. And whether benefit is added, and whether they're able to take on the broad range of things that GPs can.'* (GP*)* ^21^

#### Efficacy

When looking at efficacy and assessing if the PAs fulfilled the role they were designed for, 13 studies commented on this with mixed outcomes. The exact role PAs play in primary care has historically not been clearly defined, although NHS Employers suggest it should include taking clinical histories and examinations, seeing undifferentiated patients, and formulating diagnoses and management plans.^4^ This differs somewhat from the recent PA scope of practice documents that have been produced,^11,35^ which may lead to a change in the expected roles of PAs in the future.

Positive reviews on the outcome of PAs in primary care came from Drennan *et al*’s series of studies^23,24^ where patients felt their consultations were no different to those with GPs with high rates of satisfaction, and no significant difference between PAs' and GPs' re-consultation rates. Halter *et al's* 2017^26^ study echoed this positivity and found that patients appreciated the PA's ability to provide continuity of care and this was more important to them than the type of practitioner they saw. In Drennan *et al*'s 2017 study,^32^ the PAs were found to fulfil roles similar to GPs and other practice staff, such as nurses and nurse practitioners, and other practice staff approached PAs for advice by preference over other clinicians:

*'I think she definitely bridges the gaps* [between doctors' and nurses' work] *quite a lot and I can certainly ask, I maybe wouldn't feel as silly asking her some of the questions that I might feel a bit silly asking a doctor*.' (Practice manager)^32^

Another theme among the studies (*n* = 4) centred around the expectations and abilities of PAs, particularly those who were newly qualified.^22,27–29^ Cottrell *et al*’s 2021 study compared the expectation for PAs to undertake book-on-day appointments, long-term reviews, and results handling. They found while the majority did manage these tasks, some PAs did not meet expectations, with 86% of the PAs surveyed undertaking booking on-the-day activities by the end of the first-year internship.^28^ Nelson *et al*’s 2019 study provides some background to this, with PAs feeling less confident starting in primary care, citing the short placement time in primary care, and the independent nature of primary care work with a less structured support system than is found in hospital.^22^ Having clear expectations for the PA with realistic information on their capabilities before starting the placement was suggested as a method to combat this issue:

*'... at this stage, I wanted to be working in secondary care because, I don’t feel comfortable enough being quite so independent* [in general practice]*, straight out of medical school.' (PA*)^22^

Once again, the issue around regulation and specifically prescribing was cited as a potential problem that needs to be overcome.^23,26^

#### Cost-effectiveness

Only one study in this review attempted to quantify the relative costs of GPs and PAs, this was Drennan *et al*’s 2014 paper.^23^ They calculated mean consultation time of PAs and GPs and standardised costing for GP and PA staff to calculate cost per average consultation, which estimated PAs being £6.22 cheaper per consultation. They acknowledged that these figures do not take into account costs of GP supervision time and GP time, such as prescription signing, which may erode or remove this cost-saving,^23^ which is a considerable limitation when assessing cost-effectiveness.

Seven other papers discussed cost-effectiveness in a more qualitative manner.^21,22,24,27,28,32,36^ Some felt that the benefits outweighed the costs although these were in themselves hard to quantify.^36^ In fact the problems quantifying impact were highlighted by Nelson *et al* (2019):^22^

*'It’s anecdotal — we’re finding* [PA] *useful; but to actually quantify how many appointments she’s … taking off* [GPs] *involves an awful lot of time that we haven’t got.’* (GP)^22^

Where study participants felt PAs may not be cost-effective, this was focused around the perceived limitations and uncertainty around the role. Time spent supervising the PAs was repeatedly noted as a challenge to the cost-effectiveness,^22,36^ in addition to the inability to independently prescribe medications. Cost-effectiveness was not established when comparing PAs to other clinical practitioners such as nurses or pharmacists, with prescribing again being highlighted as a limit,^27,32^ although no studies quantitatively compared these roles directly.

Overall, it was felt to be ambiguous whether PAs would prove to be cost-effective for primary care, with differing views within the studies and only one study that had attempted to quantify this issue within limited parameters.

## Discussion

### Summary

The PA workforce is relatively new to UK primary care workforce and correspondingly there is limited published literature thus far on the topic.

Reassuringly, this review found the studies that reported on safety noted that when consultations were objectively reviewed no safety concerns were identified. This was repeated across studies in this review and with equivalent secondary care studies.^37^ Concerns were raised around clear identification of the PA role so patients are clear which professional they are consulting with.^23,28^ This correlates with the recent scope of practice documentation that has been produced by the BMA and RCGP.^11,35^

This review found that the primary role of the PA within primary care was running on-the-day urgent clinics, followed by non-urgent appointments and reviewing test results. This was both the expectation of the employers and of the PAs.^28,31^ In Cottrell *et al*’s 2021 study the majority of PAs were undertaking book-on-day appointments by the end of their first year although there were around 14% who were not^28^.The PAs were generally felt to see the less complex patients, and those with less chronic illness.^22,24,25^ PAs were felt to enhance continuity of care and this is also consistent with the studies that considered secondary care, with this being rated a key benefit to having them as part of the team.^37^ Patient views were noted to be positive in the studies and it was commented that they would be happy to see a PA in many cases if it helped maintain continuity of care.

The lack of regulation and ability to prescribe or request tests, such as radiology, were frequently mentioned across the outcomes assessed. It was seen as a limiting factor as it created transfer of workload to the prescribing clinician and created delays within the consultations themselves.^21,23,26,27^

Impact on overall practice workload by PAs was not clear, and with changes in recommended scope of practice this may need further assessment with these constraints taken into account.^35^

Finally, a crucial consideration in healthcare planning is cost-effectiveness. There were limited studies looking at this and only one that attempted to quantify these costs in 2014.^23^ This tentatively found PAs to be cost-effective compared with GPs but only accounting for the costs of the practitioners themselves and acknowledged that considering supervision and other costs may well erode this cost-effectiveness. Overall, this review has found no comprehensive assessment of the overall cost-effectiveness of PAs in UK primary care has been undertaken thus far.

### Strengths and limitations

This is the most up-to-date review focusing exclusively on studies within UK primary care, which gives it a unique evidence base compared with previous reviews. This may help inform future research priorities and decisions around PAs in the primary care workforce, particularly relevant with the planned government review into PAs and anaesthesia associates.^38^

There are, however, several clear limitations to this review. By the nature of the relatively small population available to be studied owing to the recent nature of the PA profession in the UK, the sample sizes have naturally been small. While including studies from abroad, primarily the US where PAs have been employed since the 1960s,^39^ would have broadened the evidence base, this study specifically aimed to gain insight from PA placement in UK primary care. Similarly, only primary care studies were considered to take into account the intrinsic differences between primary and secondary care. However, looking into these may provide further insight in future reviews.

It is also notable that there is a high proportion of studies (9/16) with data from over 5 years ago. When studying a relatively new profession this may have impact on how experienced PAs are within primary care, and therefore how much of a long-term view we are able to take on this cohort's impact on primary care. Similarly, with the significant expansion of the role within primary care, the nature of those practices that initially employed PAs may well be different to the much larger sample we now see.

Finally, it is noted a large number of these studies were by the same groups of authors and in fact a number were linked studies funded by an National Institute for Health and Care Research (NIHR) grant.^40^ Given the mainly qualitative nature of the studies, where author positionality can affect interpretation and analysis, this may limit the perspectives synthesised in this review.

### Comparison with existing literature

As noted, overall there is limited literature on PAs in UK primary care although a wider evidence base is available considering secondary care and international settings.

Several points were consistent in both primary and secondary care, despite their differing requirements. This included reassuring results around assessments of safety,^37^ and positive impacts on continuity of care. Public perception and openness to the role of PAs was also found to be positive, which was echoed in the secondary care studies^37^ and results from the US with a large national survey showing no difference between patient views on PAs or GPs.^41^ Clear identification of PAs was highlighted as important across settings, with Swainston *et al* (2024) in their systematic review of public perceptions of PAs in both primary and secondary care, finding PAs were often not clearly identified to patients, which led to confusion around their roles.^17^

Similar concerns have previously been identified around prescribing limitations, with a systematic review looking at PAs in secondary care by Drennan *et al* (2019) identifying this as a limiting factor as to how PAs are able to reduce doctors' workloads,^37^ indicating a consistent limitation across settings. With the recent decision to begin bringing PAs under General Medical Council (GMC) regulation in 2024,^42^ there is potential for this to change, although currently this will not allow PAs to prescribe. PAs who work the US are able to prescribe, which removes one of the major limiting factors identified in this review of UK PAs.^43^

The findings on integration of new PAs into the primary care role was also found in international studies, with Burrows *et al* (2020) noting in their study of Canadian PA integration that the nature of PA integration is non-linear and described PAs adapting to the roles as required in the specific setting, indicating flexibility was needed on both parts to utilise PAs most effectively.^44^ Taking these findings into account could help role-planning for PAs and limit the expectation and abilities gap, which were noted in this review. The findings can assist in workforce planning when considering the most effective team design with differing professionals fulfilling complementary roles to improve workload demands across the team. However, seeing on-the-day patients, which was found to be the primary role of PAs in this review, could potentially be in conflict with BMA and RCGP^11,35^ guidance around seeing undifferentiated patients if not managed correctly.

Finally, this review found no clear evidence of cost-effectiveness as assessed in UK primary care; this has noted to be a gap in evidence internationally. A review by Laurent *et al* (2009), primarily on US studies but including some UK ones, looked at cost-effectiveness of PAs, pharmacists, and nurse practitioners and found a similar deficit in evidence.^45^ However, some US studies have showed that when utilised correctly in primary care they can be cost-effective compared with physicians.^46,47^ although, as noted, they work quite differently to their UK equivalents and may not be directly comparable.

### Implications for research and practice

This review demonstrates in the first instance the need for further research into this new profession. With government plans on expansion being considered in the new review,^38^ it is crucial that evidence-based decisions are made.^38^

While general research of PAs in UK primary care is limited, we noted a distinct gap in research comparing PAs with other non-GP primary care staff such as nurse practitioners, paramedics, and pharmacists. When considering the role of PAs within primary care, this research is important to allow workforce planning, and guide policy around primary care roles moving forwards.

## Trial registration number

PROSPERO (reference: CRD42024568061)
